# Transcriptomic Analysis of Extracellular Vesicles in the Search for Novel Plasma and Thrombus Biomarkers of Ischemic Stroke Etiologies

**DOI:** 10.3390/ijms25084379

**Published:** 2024-04-16

**Authors:** Florencio J. D. M. Machado, Juan Marta-Enguita, Susan U. Gómez, Jose A. Rodriguez, José Antonio Páramo-Fernández, María Herrera, Beatriz Zandio, Nuria Aymerich, Roberto Muñoz, Rebeca Bermejo, Javier Marta-Moreno, Begoña López, Arantxa González, Carmen Roncal, Josune Orbe

**Affiliations:** 1 Laboratory of Atherothrombosis, Cima Universidad de Navarra, 31008 Pamplona, Spain; fflorenciom@alumni.unav.es (F.J.D.M.M.); jmarta@alumni.unav.es (J.M.-E.); sgomezurbae@alumni.unav.es (S.U.G.); josean@unav.es (J.A.R.); japaramo@unav.es (J.A.P.-F.); croncalm@unav.es (C.R.); 2 Instituto de Investigación Sanitaria de Navarra IdiSNA, 31008 Pamplona, Spain; mc.herrera.isasi@navarra.es (M.H.); blopez@unav.es (B.L.);; 3 Red de Investigación Cooperativa Orientada a Resultados en Salud (RICORS)-Ictus, Instituto Salud Carlos III, 28029 Madrid, Spain; beatriz.zandio.amorena@navarra.es (B.Z.); nuria.aymerich.soler@navarra.es (N.A.); roberto.munoz.arrondo@navarra.es (R.M.); jmartam@gmail.com (J.M.-M.); 4 Centro de Investigación Biomédica en Red de Enfermedades Cardiovasculares (CIBERCV), Instituto de Salud Carlos III, 28029 Madrid, Spain; 5 Hematology Department, Clinica Universidad de Navarra, 31008 Pamplona, Spain; 6 Neurology Department, Hospital Universitario de Navarra, 31008 Pamplona, Spain; 7 Neurointervencionist Radiology, Hospital Universitario de Navarra, 31008 Pamplona, Spain; rebeca.bermejo.garces@navarra.es; 8 Neurology Department, Hospital Universitario Miguel Servet, Instituto de Investigación Sanitaria de Aragón (IIS-Aragon), 50009 Zaragoza, Spain; 9 Cardiovascular Diseases Program, Cima Universidad de Navarra, 31008 Pamplona, Spain; 10 Department of Pathology, Anatomy and Physiology, Universidad de Navarra, 31008 Pamplona, Spain

**Keywords:** extracellular vesicles, ischemic stroke, biomarkers

## Abstract

Accurate etiologic diagnosis provides an appropriate secondary prevention and better prognosis in ischemic stroke (IS) patients; still, 45% of IS are cryptogenic, urging us to enhance diagnostic precision. We have studied the transcriptomic content of plasma extracellular vesicles (EVs) (*n* = 21) to identify potential biomarkers of IS etiologies. The proteins encoded by the selected genes were measured in the sera of IS patients (*n* = 114) and in hypertensive patients with (*n* = 78) and without atrial fibrillation (AF) (*n* = 20). IGFBP-2, the most promising candidate, was studied using immunohistochemistry in the IS thrombi (*n* = 23) and atrium of AF patients (*n* = 13). In vitro, the IGFBP-2 blockade was analyzed using thromboelastometry and endothelial cell cultures. We identified 745 differentially expressed genes among EVs of cardioembolic, atherothrombotic, and ESUS groups. From these, IGFBP-2 (cutoff > 247.6 ng/mL) emerged as a potential circulating biomarker of embolic IS [OR = 8.70 (1.84–41.13) *p* = 0.003], which was increased in patients with AF vs. controls (*p* < 0.001) and was augmented in cardioembolic vs. atherothrombotic thrombi (*p* < 0.01). Ex vivo, the blockage of IGFBP-2 reduced clot firmness (*p* < 0.01) and lysis time (*p* < 0.001) and in vitro, diminished endothelial permeability (*p* < 0.05) and transmigration (*p* = 0.06). IGFBP-2 could be a biomarker of embolic IS and a new therapeutic target involved in clot formation and endothelial dysfunction.

## 1. Introduction

Ischemic stroke (IS) caused by the reduction of blood flow to a part or all of the brain [1] is an important cause of mortality and morbidity worldwide, and its incidence is increasing with the aging of the population [2]. Currently, non-lacunar strokes are classified according to suspected thrombus origin in cardioembolic (CE, 35%), atherothrombotic (AT, 17%), or indeterminate or cryptogenic (45%) when the definitive cause of origin cannot be established. Among the latter, embolic stroke of undetermined source (ESUS) is a specific subtype [2]. Unravelling IS etiology is crucial for applying the most suitable therapies to patients with IS. Accordingly, several groups have proposed potential biomarkers, either circulating proteins (e.g., BNP, D-dimer, serum amyloid A, etc.), nucleic acids (CREM, ZAK, etc.), or even thrombus proteins (ATG3, RGD, GLUT-1, SERPINC1, APOA1, etc.) for this purpose [3,4,5]. However, none of these biomarkers are currently used in clinical practice, highlighting the need for novel approaches to address this gap.

The implementation of mechanical thrombectomy has improved reperfusion rates, thus becoming a cornerstone in the management of IS patients with large vessel occlusion. Moreover, it enables the study of morphological and physical characteristics of the thrombus, providing valuable insights into stroke etiology [6]. In this context, the study of circulating extracellular vesicles (EVs) as components of the liquid biopsy after IS might also represent an opportunity to characterize the molecular processes behind each IS subtype. EVs are particles released from cells that are delimited by a lipid bilayer, cannot replicate on their own, are involved in cell-to-cell communication, and contain RNAs, proteins, and lipids from the cell of origin [7]. Due to their capacity to exchange material from parent cells to others, EVs can regulate gene expression in targeted cells and modulate their function in physiological or pathological conditions [8,9]. Moreover, advancements in high-throughput RNA sequencing methods allow for the study of the transcriptomic content within circulating EVs [10]. The total number of EVs is increased in different cardiovascular diseases, including IS [11], and in association with risk factors such as hypertension, diabetes, vascular dysfunction, and atherosclerosis [11,12]. Regarding IS, Wang et al. described that the concentrations of total EVs, as well as EV subtypes (endothelial, platelet, erythrocyte, leukocyte, and monocyte derived) were significantly increased in IS patients compared to healthy controls [13].

We hypothesized that the transcriptomic analysis of circulating EVs might help to delineate the molecular profile and potential biomarkers for IS of different origins, thereby guiding clinicians in the etiological diagnosis and the preventive therapeutic strategy for each patient to prevent IS recurrences.

## 2. Results

### 2.1. Plasma EVs Characterization

**Figure 1:**
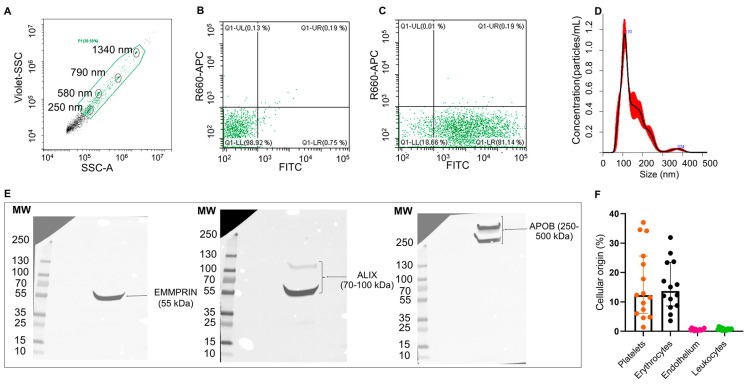
Characterization of plasma EVs from IS patients: Flow cytometry plots for isolated EVs (**A**–**C**). EV working gate was defined using the violet side scatter (violet-SSC) against the regular SSC using calibrated beads (circled in black) ranging in size from 250 nm to 1340 nm (**A**). Flow cytometry showing representative scatter plots of unstained (**B**) and CFSE-stained (**C**) EVs from IS patients within the working gate. Representative EV size distribution histogram obtained by nanoparticle tracking analysis (NTA) in IS patients (mean [SD]: 130.4 [±2.2] nm, *n* = 3) (**D**). Western blot for the EVs markers EMMPRIN and ALIX, and major plasma contaminants (APOB100) (**E**). Quantification of plasma EVs subpopulations by flow cytometry using specific antibodies against CD41/61 (platelets), CD235a (erythrocytes), CD62E (endothelial cells) and CD11b (leukocytes). Data are presented as percentage of total events (*n* = 15) (**F**).

EVs from the plasma of patients with IS (*n* = 3) were able to uptake and process CFSE dye (Figure 1A–C) and were heterogeneous in size distribution (mean [SD]: 130.4 [±2.2] nm and a mean concentration of 6.04 × 10^9^ particles/mL, Figure 1D). Moreover, isolated EVs (*n* = 1) displayed EMMPRIM and ALIX as specific EV markers and APOB100 as a coprecipitated contaminant (Figure 1E and Figure S1). Flow cytometry analysis (*n* = 15) of the cellular origin of EVs unveiled that they were mainly from platelet and erythrocyte origin (Figure 1F).

### 2.2. Transcriptomic Analysis of Plasma EVs from IS Patients

The transcriptomic analysis of plasma EVs was performed on 21 IS patients comprising all recruited AT (*n* = 6), 10 CE, and five ESUS from the first 50 recruited patients (Table S1). These patients presented a median age of 76 years, and the cardiovascular risk factor with the highest frequency was hypertension (62%). Nearly 10% of the patients had a previous IS, and 62% had severe NIHSS scores at admission. Complete recanalization (mTICI score 2b-3) after thrombectomy was achieved in 95%, and almost 43% presented 3-month functional independence.

**Figure 2:**
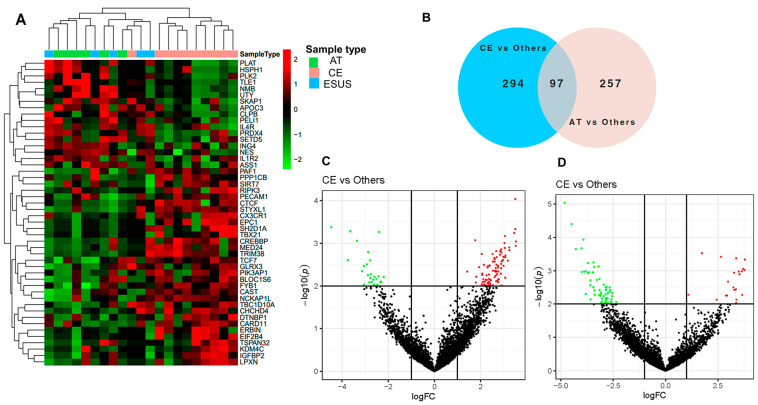
Bioinformatics analysis on plasma EVs of IS patients after RNAseq. (**A**) Hierarchical clustering and heatmap of the differentially expressed genes (DEG) in plasma EVs taking into account the IS etiologies [CE (*n* = 10, in pink), AT (*n* = 6, in green), and ESUS (*n* = 5, in blue)]. Samples are arranged in columns, and genes are presented in rows. Upregulated expression is shown in red, and downregulated expression is shown in green. The heatmap was generated using counts per million expression values (CPM, logarithmically transformed). (**B**) Venn diagrams showing DEG between etiologies (AT vs. others, and CE vs. others). (**C**,**D**) Volcano plots showing the DEG of the contrasts performed by Limma Voom for AT vs. other etiologies and CE vs. others. In red, genes with a fold-change (log2) and *p*-value (log10) higher than 1 and 2, respectively. In green, genes with a fold-change (log2) and *p*-value (log10) lower than −1 and 2, respectively.

The RNASeq analysis of EVs detected clusters of differentially expressed genes (DEG) related to IS etiologies. The heatmap in Figure 2A shows that the expression of certain genes was consistently increased or decreased for AT and CE strokes, while some ESUS mirror the expression pattern of AT and others of the CE etiology. In total, we identified 745 DEG among the study groups (*p* < 0.05), from which 391 were differentially expressed in EVs from the plasma of CE patients compared to others (AT and ESUS) and 354 in AT patients compared to others (CE and ESUS), as shown in the Venn diagram (Figure 2B) and the volcano plots (Figure 2C,D).

**Figure 3:**
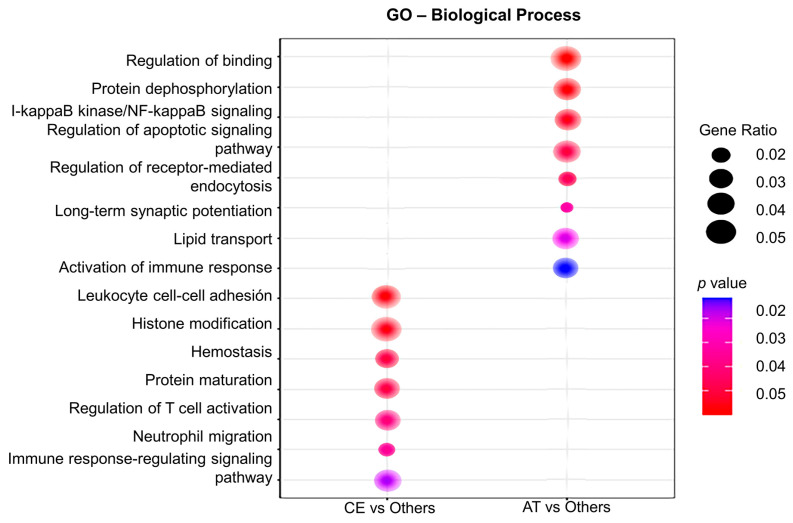
Gene ontology (GO) analysis of the differentially expressed genes in plasma EVs depending on IS etiology. The GO analysis for biological processes identifies deregulated pathways between studied groups (in the left column, CE vs. others; in the right column, AT vs. others). Node size is proportional to gene ratio, while the color spectrum indicates the level of association, ranging from weak in blue to strong in red.

Gene ontology (GO) analysis for biological processes was performed to assign gene sets to the DEG identified in CE and AT EVs. As such, plasma EVs from CE patients were enriched in transcripts involved in leukocytes cell–cell adhesion (IGFBP-2, PECAM-1), hemostasis (PF4V1, PF4), T cell activation (IGFBP-2), neutrophil migration (PECAM-1), axonal regeneration (PTEN, MAP2K1), and cardiomyocyte proliferation (PTEN, Figure 3). In contrast, in AT patients, the main enriched pathways were related to regulation of binding (RIPK2, NECTIN-2), lipid transport (APOC3), I-kappaB kinase/NF-kappaB signaling (RIPK2), the regulation of synaptic long-term potentiation (PTEN), and the activation of the immune response (CREBBP), among others (Figure 3).

Based on the DEG between IS etiologies and its association with cardiovascular diseases in previous studies, we selected three over-represented genes in EVs for further validation; namely, IGFBP-2 and PECAM-1 increased in EVs from CE patients, and NECTIN-2 overexpressed in AT patients (Supplementary Material Table S2). IGFBP-2 has been proposed as an independent predictor of cardiovascular disease severity and mortality in patients with pulmonary hypertension and heart failure [14,15], while NECTIN-2 and PECAM-1 have been associated with leukocyte endothelial transmigration and atherosclerosis [16,17], and the latter also with IS severity [17].

### 2.3. Expression of Selected Candidates in Plasma EVs by qPCR and Western Blot

**Figure 4:**
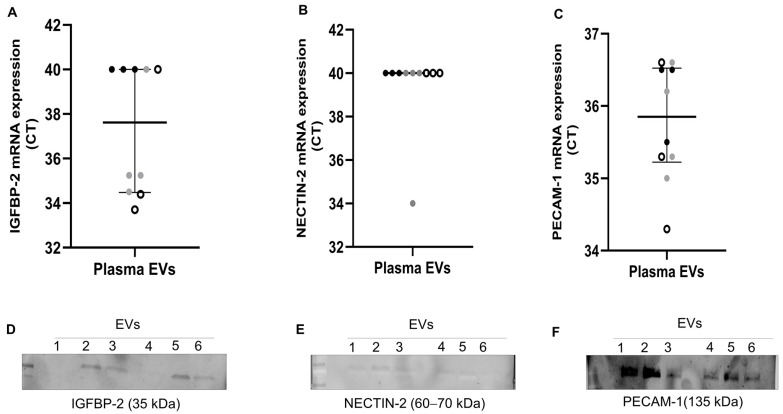
Gene expression and western blot of selected candidates in plasma EVs from IS patients. (**A**–**C**) mRNA levels, presented as Ct values, of IGFBP-2, NECTIN-2, and PECAM-1 assessed using RT-qPCR in plasma EVs from *n* = 10 IS patients (AT = 3: black dots, CE = 4: grey dots, and ESUS = 3: white dots). Undetected transcripts were assigned Ct = 40. (**D**–**F**) Western blot of plasma EVs from IS patients (*n* = 3 AT and 3 CE) showing selected candidates and their expected molecular weights (MW). EVs 1 to 3 are from AT patients, and EVs 4 to 6 are from CE patients.

To further validate the presence of IGFBP-2, NECTIN-2, and PECAM-1, we performed RT-qPCR (*n* = 10) and western blot on plasma EVs (*n* = 3 AT and 3 CE) of the same patients. As shown in Figure 4A–C, IGFBP-2 was exclusively detected in ESUS and CE EVs (*n* = 3 and 4, respectively), but not in AT EVs (*n* = 3). PECAM-1 was detected in all patients’ EVs (average Cts: AT = 35.3, CE = 35.7, ESUS = 35.4), whereas NECTIN-2 was only detected in a single sample. Next, we assessed whether proteins encoded by selected genes were also encapsulated in EVs. We were able to detect the proteins encoded by our candidates in plasma EVs of IS patients (*n* = 3 AT and 3 CE) through western blot analysis (Figure 4D–F and Figure S2).

### 2.4. Circulating Levels of the Proteins Encoded by the Selected Genes

The circulating levels of IGFBP-2, NECTIN-2, and PECAM-1 were measured in our cohort of 114 patients with IS, including 56% of CE, 23% of AT, and 21% of ESUS. Table 1 summarizes the clinical and demographic characteristics of our cohort that presents a median age of 75 years with 44% females, whilst most of the patients were men in the AT etiology. Hypertension was the highest prevalent risk factor (67%), and almost 58% of patients had severe NIHSS scores at admission. Almost all patients achieved complete recanalization (mTICI score 2b-3). Functionally independent patients and those who died within 3 months after IS represented 55% and 20%, respectively. 

**Figure 5:**
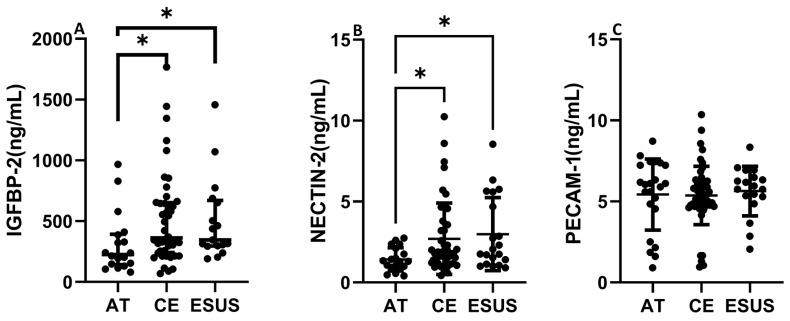
Circulating levels of IGFBP-2, NECTIN-2, and PECAM-1 according to patient clinical etiology. IGFBP-2 and NECTIN-2 concentrations were increased in CE and ESUS patients compared to AT (**A**,**B**). No differences were observed in blood levels of PECAM-1 according to IS origin (**C**). (*n* = 19–23 AT, *n* = 40–54 CE and *n* = 19–23 ESUS). * *p* < 0.05 vs. AT.

IGFBP-2 was significantly increased in serum from CE and ESUS patients compared to those with AT etiology (median [IQR]: 395.7 [250.4–672.6] ng/mL CE vs. 226.1 [142.8–393.0] ng/mL AT, *p* < 0.05) and vs. 346.6 [295.7–670.2] ng/mL ESUS, *p* < 0.05, Figure 5A). In contrast to the RNAseq data, NECTIN-2 was also significantly increased in CE and ESUS compared to AT patients (median [IQR]: 1.8 [1.2–3.3] ng/mL CE vs. 1.3 [0.9–2.3] ng/mL AT, *p* < 0.05 and 2.1 [1.3–4.9] ESUS, *p* = 0.05, Figure 5B), while no differences were observed for PECAM-1 (Figure 5C).

To further study the potential association of IGFBP-2 and NECTIN-2 with the clinical characteristics of patients with IS, Pearson correlation test for quantitative variables and Mann–Whitney U test/Student’s *t* test for qualitative parameters were performed (Supplementary Table S3). IGFBP-2 was correlated with age (r = 0.20, *p* = 0.01) and baseline NIHSS (r = 0.33, *p* = 0.003). Additionally, IGFBP-2 was associated with AF (*p* = 0.02) and functional dependence (*p* = 0.03) and tended to increase in anticoagulated patients (*p* = 0.08), but not in fibrinolytic treated patients. Despite this observation, patients with CE, IS, and AF on anticoagulants presented similar levels of IGFBP-2 when compared with patients with AF not receiving anticoagulants [median [IQR]: 572.6 [277.2–1080.5] vs. 459.0 [319.4–761.7], *p* = 0.87]. In contrast, no association was observed between NECTIN-2 and the studied parameters (Table S3).

In a binary regression model, circulating levels of IGFBP-2 showed an independent association with embolic etiology in both univariate [odds ratio, OR (95% confidence interval, CI) 0.99 (0.99–1.00) *p* = 0.04] and multivariate analyses after adjusting for age, sex, and baseline NIHSS [OR (95%CI) 0.99 (0.99–1.00), *p* = 0.03]. Conversely, the association between NECTIN-2 and embolic etiology did not retain significance after adjusting for age, cardiopathy, previous IS, and statin therapy [OR (95%CI) 1.70 (0.93–3.10), *p* = 0.08].

Based on our results, we focused on the potential role of IGFBP-2 as a biomarker of IS diagnosis (embolic vs. AT). Univariate ROC curve analysis revealed a significant association between IGFBP-2 and embolic IS compared to AT etiology [AUC (95% CI) 0.71 (0.57–0.84), *p* = 0.007]. The optimal cutoff for IGFBP-2 according to the Youden index was 247.6 ng/mL, with 77.8% sensitivity and 61.1% specificity.

Binary logistic regression analyses with the defined cutoff showed that patients with IGFBP-2 > 247.6 ng/mL had a five- to eightfold higher probability to have an embolic (CE or ESUS) stroke in both univariate [OR (95%CI) 5.50 (1.7–16.8), *p* = 0.003] and multivariate models [OR (95%CI) 9.51 (2.13–42.54), *p* = 0.003] after adjusting for age and sex (model 1), or [OR (95%CI) 8.70 (1.84–41.13), *p* = 0.003] when baseline NIHSS was included in model 2 (Table S4).

In an independent cohort of hypertensive patients (Table S5), those with AF (*n* = 78) had significantly higher circulating levels of IGFBP-2 compared to those without AF (*n* = 20) (median [IQR]: 438.5 [337.5–565.6] ng/mL vs. 115.7 [97.1–164.0] ng/mL, *p* < 0.001). Interestingly, the levels of IGFBP-2 in hypertensive patients with concomitant AF, were similar to the IGFBP-2 levels in CE stroke patients.

### 2.5. Expression of IGFBP-2 in Thrombus of Patients with IS and in Left Atrium of Patients with AF

**Figure 6:**
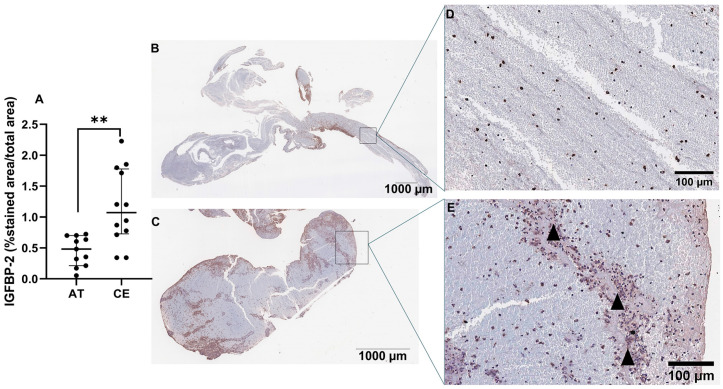
IGFBP-2 immunostaining in IS thrombi. Quantification of IGFBP-2 immunostaining in thrombi from IS patients (*n* = 11 AT, *n* = 12 CE) (**A**). Representative images of AT (**B**) and CE (**C**) thrombi and their respective magnifications (**D**,**E**). Arrowheads represent the interfaces rich in platelets and fibrin. Scale bars represent 1000 µm (**B**,**C**) and 100 µm (**D**,**E**). ** *p* < 0.01 vs. AT.

Next, we assessed the presence and location of IGFBP-2 protein in IS thrombi (*n* = 11 AT, *n* = 12 CE). As shown in Figure 6A, IGFBP-2 expression was significantly increased in thrombi from CE origin compared with AT (*p* < 0.01). IGFBP-2 was found in peripheral regions, rich in inflammatory cells (CD68+, CD45 and NE+ cells, Figure S3) and in the interfaces rich in platelets and fibrin (Figure 6B–E). The IGFBP-2 thrombus expression was increased in patients taking anticoagulants (*p* = 0.03) and reduced in those who died during the 3 months of follow-up (*p* = 0.05) (Table S6).

Given that AF is a major cause of CE stroke, we further analyzed IGBP-2 expression in the left atria of patients with AF. IGFBP-2 staining was evident in the atria of patients with AF mainly localized in cardiomyocytes, as well as in endothelial and mesothelial cells (Figure S4).

### 2.6. Biological Function of IGFBP-2 in Endothelial Cells and Clot Formation

**Figure 7:**
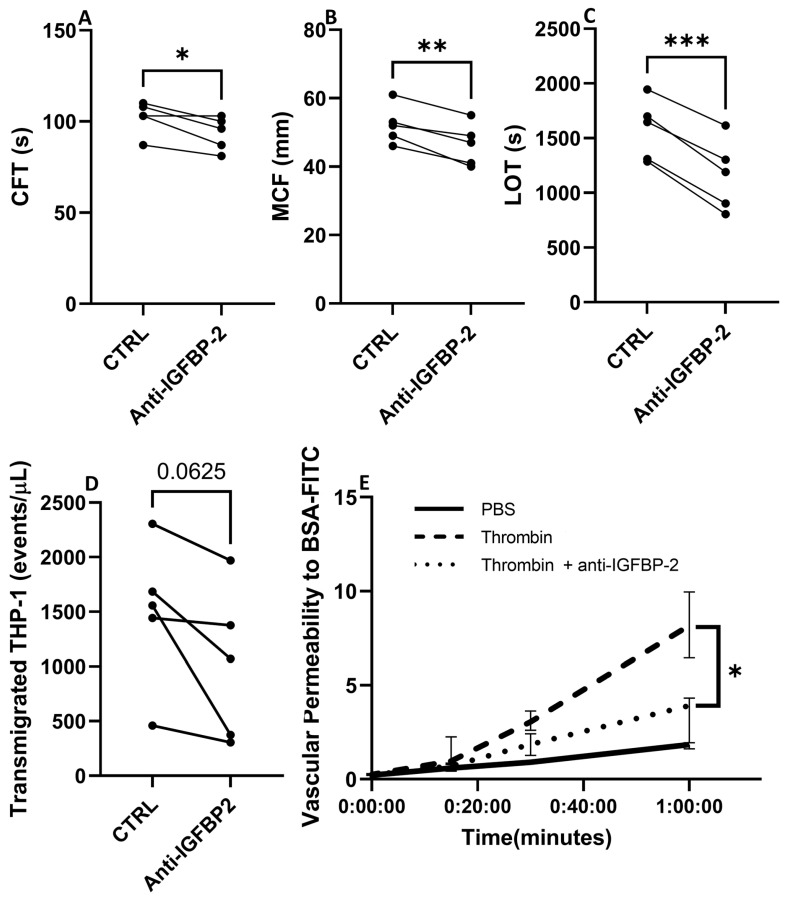
Biological impact of blocking IGFBP-2 in clot formation and endothelial cell function. Changes in hemostatic parameters after IGFBP-2 blockade in thromboelastometry assays with blood samples of healthy subjects (*n* = 5) (**A**–**C**). CFT: Clot formation time, MCF: maximum clot firmness, LOT: lysis onset time. * *p* < 0.05, ** *p* < 0.01 and *** *p* < 0.001 vs. ctrl (*n* = 5/group). Number of transmigrated THP-1 monocytes in presence or absence of a neutralizing antibody against IGFBP-2 across TeloHAECs (*n* = 5/group) (**D**). Vascular permeability to BSA over time in hCMEC/D3 control cells (black solid line), or thrombin stimulated cells with (dotted line) or without (dashed line) a pretreatment with a neutralizing IGFBP-2 antibody (**E**). Four independent experiments were performed, with two technical replicates in each one. * *p* < 0.05 vs. thrombin at 1 h.

The pathophysiology of CE stroke is related to endothelial dysfunction and coagulation activation, processes in which IGFBP-2 has been involved [18,19,20]. Therefore, to explore the role of IGFBP-2 in CE IS, we performed ex vivo and in vitro experiments in whole blood and endothelial cells with an IGFBP-2 neutralizing antibody. As presented in Figure 7A–C, the blockade of IGFBP-2 in ROTEM assay performed in blood samples of healthy volunteers led to significant changes in clot characteristics. It is noteworthy that IGFBP-2 blockage slightly shortened the clot formation time (*p* < 0.05) but markedly decreased clot firmness (*p* < 0.01) and the lysis onset time (*p* < 0.001).

Finally, the inhibition of endothelial IGFBP-2 decreased the number of transmigrated leukocytes (*p* = 0.06, Figure 7D) and reduced BSA permeability induced by thrombin at 1 h by 83% (*p* < 0.05 vs. thrombin, Figure 7E).

## 3. Discussion

In this study, we performed, for the first time, a transcriptomic analysis of plasma EVs from patients with IS, revealing important insights related to EV composition and biological pathways involved in IS etiologies. Following the transcriptional analysis of plasma EVs, three potential candidates for IS etiology were identified, including IGFBP-2, NECTIN-2, and PECAM-1. Among these, IGFBP-2 stood out not only as a potential circulating biomarker of embolic stroke etiology but also as a regulator of clot formation and lysis and endothelial function.

EVs are components of liquid biopsy shed from all cell types into body fluids and represent a sensitive and noninvasive approach for diagnosis, the identification of therapeutic targets, real-time disease tracking, and the monitoring of treatment effects [21]. As part of EV characterization, we performed flow cytometry to determine the levels of EVs from different cellular origins. In agreement with previous data and reflecting the distribution of blood cells [22], we found a predominance of platelet- and erythrocyte-derived EVs in IS patients, followed by the much less abundant leukocyte and endothelial EV subpopulations. Variations in EV levels, either from brain, vascular wall, or blood origin, may represent potential valuable biomarkers for stroke that can mirror in blood the processes occurring in the brain, as well as in other organs. As such, increased levels of platelet, endothelial, erythrocyte, leukocyte, and monocyte EVs have been reported in post-acute IS compared with subjects without vascular disease [13,22]. In addition, in most studies, the increase in platelets and endothelial derived EVs has been further correlated with stroke severity and long-term clinical outcome, although the benefits of measuring platelet EV levels still remains controversial when considering antiplatelet treatment [23].

The content of EVs, including RNAs, proteins, etc., depends on the cellular activation status. Therefore, the analysis of the transcriptional content of EVs post-IS could be a valuable tool for identifying novel biomarkers for IS etiology and prognosis. The bioinformatics analysis of the DEG in plasma EVs from patients with CE IS revealed the enrichment in gene sets related to the hemostatic system (PF4V1, PF4), the immune/inflammation function, e.g., T cell activation and differentiation (IGFBP-2), cell adhesion (IGFBP-2, PECAM-1), or migration (PECAM-1), as well as in molecular processes related to axonal regeneration (PTEN) and cardiomyocyte cell proliferation (PTEN). Consistent with our findings, other authors reported an enrichment in molecular processes related to cardiomyocyte death, implicating PTEN and inflammation, including AQP9, PTEN, or SMARCA4 (recognized for its involvement in heart fibrosis), in the blood of CE stroke patients [24]. In the analysis of AT EVs, we found DEG primarily linked to protein binding (RIPK2, NECTIN-2), lipid catabolism (APOC3, NECTIN-2), the I-kappaB kinase/NF-kappaB pathway (RIPK2), and the activation of the immune response (CREBBP). Notably, a microarray analysis of blood samples from AT patients revealed a deregulation of genes related to inflammation (ADA, ADORA3, ADRB2, ALOX5AP) [24], cellular migration (MMP9, FASLG, CX3R1, RAG1, TNF, IRAG1, CX3CR, and THBS1), and lipid deposition during the atherosclerotic process [24,25]. Although we found a slight discrepancy compared to previous data showing an enrichment of hemostasis gene set in AT etiology [24], this difference could be explained, at least in part, by the involvement of the hemostatic system in the pathophysiology of both CE and AT strokes. Acute coagulation and fibrinolytic activation have been previously proposed in patients with CE stroke [26]. Additionally, cardiovascular risk factors, valvular heart disease, coronary artery disease, congestive heart failure, chronic kidney disease, and inflammatory disorders have been established as thrombogenic substrates for both AF and IS. This vascular substrate is thrombogenic through two mechanisms, including (1) via atrial cardiomyopathy and subsequent AF, and (2) through non-atrial mechanisms, such as atherosclerosis of the large arteries or medium vessel disease, which are causes of AT stroke [27].

Among the DEG in CE and AT EVs, IGFBP-2, PECAM-1, and NECTIN-2 stood out as promising candidates for IS etiology. IGFBP-2 has been found to be expressed in the brain, contributing to neurotrophic and regenerative functions [28], as well as in the heart [29] and the serum of patients with heart failure [19,30]. PECAM-1 has been involved in the pathophysiology of inflammation, coagulation, and atherosclerosis [17,31], and NECTIN-2 has been associated to atherogenesis [32]. These findings suggest a possible role for any of these candidates in the context of IS. Hence, in addition to corroborating their presence in EVs using other methods, such as qPCR and western blot, we assessed their circulating levels using ELISA. Despite the initial expectations, PECAM-1 levels were similar among IS groups. In contrast to RNAseq data, blood NECTIN-2 was higher in CE and ESUS patients vs. AT and was not able to predict IS etiology in presence of other confounding factors, indicating a weak association of NECTIN-2 with IS etiology. However, circulating levels of IGFBP-2 were similarly increased in CE stroke, consistent with the observation of EVs in the RNASeq analysis and significantly associated with IS etiology in the multivariate analysis. This strengthens its potential role as biomarker of IS etiological diagnosis. Additionally, we observed increased circulating IGFBP-2 in patients at high risk of CE stroke, such as those with AF. In this particular context, IGFBP-2 has been previously linked to cardiovascular diseases that might clinically present with AF, including heart failure and cardiomyopathy [14,15,33]. This association is likely attributed to the ability of IGFBP-2 to modulate IGF1 [14], which in turn, promotes cardiomyocyte proliferation [34], left ventricular remodeling, and hypertrophy [35], thereby influencing left ventricular contractility and cardiac function [14,36]. Moreover, IGFBP-2 levels have recently been directly and independently associated with an increased risk of incident AF in adults in a prospective proteomic study involving over 15,000 subjects followed for 6 years [37]. In this regard, the identification of ESUS patients with elevated levels of IGFBP-2 might have clinical implication, as this subgroup of ESUS patients might benefit from anticoagulation treatment [38].

To dissect the role of IGFBP-2 in IS, also at a local level, we performed immunohistochemistry in IS thrombi. Similar to EVs and plasma, CE thrombi presented increased expression of IGFBP-2 compared to AT thrombi, being localized in peripheral regions rich in inflammatory cells, as well as in the interfaces rich in platelets and fibrin, suggesting a possible role in thrombus formation/consistency/lysis. These data led us to explore its possible role in clot formation and lysis using a ROTEM assay. The blockade of IGFBP-2 in clotting experiments shortened clot formation time, reduced clot firmness, and decreased lysis onset time. IGFBP-2 has been previously associated with the reduction of platelet activation and aggregation through its role as an IGF-1 scavenger, reducing IGF-1-dependent co-stimulation for platelet activation [20]. Therefore, blocking IGFBP-2 might partially justify the observed shortening in clot formation time by enhancing platelet activation through increasing the bioavailability of IGF-1. Unexpectedly, a decrease in clot firmness and the early onset of lysis was observed in ROTEM tests after blocking IGFBP-2, suggesting that IGFBP-2 might have additional coagulation functions that are independent of IGF-1-induced platelet activity. As reported for IGFBP-3, IGFBP-2 might also be capable of binding to fibrinogen and fibrin through the common heparin binding domain [39] and could influence the polymerization of fibrin fibers and clot firmness. We hypothesize that the inhibition of IGFBP-2 might reduce fibrin crosslinking, thereby decreasing clot firmness and allowing greater access of fibrinolytic therapy. However, future studies are needed to provide a more comprehensive understanding of the involvement of IGFBP-2 in hemostasis and coagulation.

Based on the increased levels of IGFBP-2 in patients with hypertension and AF, and the data from other authors showing the expression of IGFBP-2 in hearts of rats with heart failure [36], we performed immunohistochemistry to determine the expression and localization of IGFBP-2 in the left atria of patients with AF at high risk of CE stroke. IGFBP-2 was mainly observed in cardiomyocytes and endothelial and mesothelial cells. Taking in consideration that the endothelial expression of adhesion molecules, chemotactic agents, and growth factors results in higher thrombogenicity of the atrium [40], and that IGFPB-2 has been related to angiogenesis [18], vascular permeability, and transendothelial migration processes [18,31], we performed in vitro experiments to determine the effect of IGFBP-2 neutralization on endothelial function. IGFBP-2 neutralization almost significantly diminished the transendothelial migration of monocytes and significantly decreased microvascular permeability that could contribute to reduced thrombo-inflammation and thrombogenicity. Our results suggest a possible beneficial effect of IGFBP-2 blockage in the context of CE stroke modulating endothelial dysfunction.

This study has certain limitations. Firstly, the total number of samples was limited, specifically in the case of AT IS samples, which are less prevalent compared to CE IS. Secondly, the RNA content of EVs is scarce, thereby compromising the sensitivity of conventional PCR in detecting specific transcripts within EVs. Thirdly, while ROTEM functional tests were conducted to assess how IGFBP-2 inhibition affects ex vivo clot formation and lysis, this technique primarily mirrors the generation of static, low-flow thrombi, which aligns more closely with venous conditions rather than arterial thrombosis. Moreover, it should be noted that the results of this study are for the generation of new hypotheses for further study and that a larger sample size should be used in future validation studies.

## 4. Materials and Methods

### 4.1. Study Cohorts

IS patients: One hundred and fourteen (*n* = 114) patients diagnosed with large vessel occlusion (LVO) of AT, CE, and ESUS etiologies and treated with endovascular thrombectomy (EVT) were included. Among IS patients, 96 were from the Stroke Unit of the Hospital Universitario de Navarra (ref. 84/2018), recruited between November 2015 and January 2023, and 18 were from Miguel Servet University Hospital (ref. 16/2021). For all participants in the study, a previous written informed consent was obtained directly from them or from an authorized person. Samples and data from patients included in the study were provided by the Biobank of the University of Navarra and the Aragon Health Sciences Institute in the framework of the Biobank of Aragon and were processed following standard operating procedures with the approval of the Ethical and Scientific Review Boards. The decision to perform one or both IS treatments (mechanical treatment and/or intravenous tissue-type plasminogen activator (tPA)) was made following current international guidelines [41]. In these patients, a detailed medical record, including age, sex, history of cardiovascular disease, cardiovascular risk factors, glucose level at admission, AF before or during the 3 months after the event, the use of antithrombotic drugs (antiplatelet agents and anticoagulants), and thrombolytic treatment with tPA, was registered. IS severity was assessed by the National Institutes of Health Stroke Scale (NIHSS). Cardiovascular risk factors included (1) hypertension, defined as patients taking antihypertensive drugs or with blood pressure > 140/90 mmHg on repeated measurements, (2) type-2 diabetes, defined as patients on antidiabetic drugs, fasting blood sugar ≥ 126 mg/dL or HbA1c ≥ 6.5%, or a casual plasma glucose > 200 mg/dL, (3) hyperlipidemia, defined as patients receiving lipid-lowering drugs or with an overnight fasting cholesterol ≥ 240 mg/dL, triglycerides ≥ 200 mg/dL, or low-density lipoprotein (LDL) cholesterol ≥ 160mg/dL, and (4) current smoking habits. We used the Trial of Org 10172 in Acute Stroke Treatment (TOAST) for the etiological classification of IS patients [1]. To assess the recanalization rate after endovascular therapy (EVT), an angiographic study was conducted, and the modified thrombolysis in cerebral infarction (mTICI) score was applied. A successful recanalization was defined as mTICI 2b, 2c, or 3 [42].

To establish the 90-day modified Rankin scale (mRS) score, a face-to-face interview with a neurologist specialized in stroke was performed to determine the following clinical outcomes: (a) 3-month all-cause mortality and (b) 3-month functional independence, defined as 90-day mRS < 3 [43].

Hypertensive patients: An independent cohort of 98 hypertensive patients was recruited from the Clinica Universidad de Navarra (ref. 134/2014 and 140/2014) without atrial fibrillation (AF, *n* = 20) or with AF (*n* = 78).

### 4.2. EV Separation from Platelet-Free Plasma

Blood samples were collected in citrated tubes (Greiner Bio-one, Madrid, Spain) and processed within 3.5 h after mechanical thrombectomy. Citrated blood samples underwent two sequential centrifugations (Allegra X-30, Beckman Coulter, Brea, CA, USA), the first at 2000× *g* for 10 min at room temperature (RT) and the second at 2500× *g* for 15 min at RT. The obtained platelet-free plasma (PFP) was aliquoted and stored at −80 °C.

EVs were isolated from 400 μL of PFP diluted in 800 μL of filtered PBS (1 × 0.22 µm, followed by 1 × 0.1 µm filter) and centrifuged at 20,000× *g* for 70 min at 4 °C (Mikro 22R, Hettich Zentrifugen, Tuttlingen, Germany). The supernatant was carefully removed, and the pellet was homogenized in filtered wash buffer (10 mM HEPES, 0.9% NaCl and 7.4 pH). A second centrifugation was performed (20,000× *g* for 70 min at 4 °C), and the pellet was resuspended in 100 μL of filtered PBS and stored at −80 °C.

### 4.3. EV Characterization Using Nanoparticle Tracking Analysis (NTA) and Flow Cytometry

Nanoparticle tracking analysis: The characterization of the EV particle size distribution and concentration was performed using nanoparticle tracking analysis (NTA) according to the manufacturer’s instructions (NanoSight NS300, Malvern Instruments Limited, Malvern, UK) in *n* = 3 isolated EV samples.

Flow cytometry: To assess carboxyfluorescein N-succinimidyl ester (CFSE, Sigma-Aldrich, St. Louis, MO, USA) uptake by EVs, nanoscale flow cytometry was performed (*n* = 3). A total of 10 μL of isolated EVs were labeled with 0.2 mM CFSE for 30 min at 37 °C and washed with 450 μL of wash buffer (2% BSA, 10 mM HEPES, 0.9% NaCl and 7.4 pH) for 70 min at 20,000× *g* (Mikro 22R, Hettich Zentrifugen). The supernatant was carefully removed, and the pellet was diluted in 100 μL of PBS (filtered 1 × 0.22 µm, followed by 1 × 0.1 µm). To determine EV cellular origin (*n* = 15), 5 µL of EVs were labeled with specific antibodies, including 20 µg/mL PE antihuman CD62E for the endothelium, (clone HCD62E, Biolegend, London, UK), 1 µg/mL APC antihuman CD41/61 for platelets (clone A2A9/6, Biolegend), 1:10 dilution PC7 antihuman CD11b (clone Bear1, Beckman Coulter) for leukocytes, and 5 µg/mL FITC antihuman CD235a for erythrocytes (clone KC16, Beckman Coulter). Isotype control antibodies PE mouse IgG2a,ĸ (clone MOPC-173, Biolegend), APC mouse IgG2a,ĸ (clone MOPC-173, Biolegend), PC7 mouse IgG1 (clone 679.1Mc7, Beckman Coulter), and FITC mouse IgG1,ĸ (clone MOPC-21, BD) were used as negative controls. Antibodies and corresponding isotype controls were diluted in PBS, filtered two times, and centrifuged for 5 min at 18,000× *g* (Mikro 22R, Hettich Zentrifugen) before labeling the EVs (20 min, at room temperature (RT) in darkness). Flow cytometry was performed on CytoFlex (Beckman Coulter). To define the analysis gate, we used calibrated polystyrene beads of sizes 0.25, 0.58, 0.79, and 1.34 μm (Spherotech, Davis, CA, USA). The results were analyzed with CytExpert 2.3 software (Beckman Coulter).

### 4.4. Western Blot

Specific EV protein markers and selected candidates were assessed in EVs obtained from PFP samples (*n* = 3). A total of 12.5–20 μL of isolated EVs were lysed using thermal shock (3× switch from 37 °C to liquid N2). The obtained proteins were loaded in SDS-PAGE gels (4–20% Mini-PROTEAN TGX Stain-Free, Bio-Rad, Hercules, CA, USA) and transferred onto nitrocellulose membranes (Trans-Blot Turbo, Bio-Rad). The blots underwent an overnight incubation with primary antibodies, including 0.125 μg/mL mouse anti-AIP1/Alix (clone 49/AIP1, #611620, BD Bioscience, Mississauga, ON, Canada), 1 μg/mL mouse monoclonal antihuman EMMPRIN/CD147 (clone IT10C5, #21451471, Immunotools, Friesoythe, Germany), 1 μg/mL goat polyclonal antihuman apolipoprotein B100 (AF3260, Novus Biologicals, Centennial, CO, USA), 5 µg/mL antihuman NECTIN-2 (MA5-34802, Invitrogen, Carlsbad, CA, USA), 2 µg/mL antihuman PECAM-1 (M0823, Dako, Carpinteria, CA, USA ), and 1:500 dilution antihuman IGFBP-2 (MA5-15400, Invitrogen). Peroxidase-conjugated secondary antibodies (1:5000 and 1:10,000 dilution) were applied as needed. Peroxidase activity was detected with a chemiluminescent substrate (TMA-6, Lumigen, Southfield, MI, USA), and images were acquired with the Chemidoc imaging system (Bio-Rad).

### 4.5. RNA-Seq Library Construction

RNA-Seq was conducted in EVs isolated from PFP (*n* = 21) of IS patients from different etiologies according to the TOAST classification [CE (*n* = 10), AT (*n* = 6) and ESUS (*n* = 5)] as previously described [44,45]. Briefly, plasma EVs were separated using sequential centrifugation and resuspended in 100 μL of lysis/binding buffer (Invitrogen). RNA transcripts were captured with Dynabeads oligo (dT) (Invitrogen) and reverse transcribed. RNA samples underwent a linear amplification using in vitro transcription, followed by a fragmentation into 250–350 bp. Partial Illumina adaptor sequences were used, and a second reverse transcription reaction was performed. Full Illumina adaptor sequences were successfully added, and the libraries were sequenced (NextSeq 2000, Illumina, San Diego, CA, USA).

### 4.6. Bioinformatic Analysis

The quality of the data was evaluated with FastQC software (v0.11.8), and the processing of the reads was carried out using Trimmomatic (v0.38) [46]. The resulting reads were aligned with STAR using GRCh38 human assembly and Gencode v38 as a genome annotation reference [47]. Then, duplicated reads were removed by applying UMI-tools dedup function [48]. Finally, expression levels were calculated with featureCounts software (v1.6.0) [49]. The obtained gene expression data was normalized using the analysis pipeline available in LIMMA package (TMM normalization and logCPM calculation using Limma voom method) [50]. After the quality assessment and outlier detection with R/Bioconductor [51], a filtering process was performed. Genes with read counts lower than 6 in more than the 50% of the samples were excluded. LIMMA was used to identify genes with significant differential expression. The selection of differentially expressed genes was based on a *p*-value cutoff (*p* < 0.05), and further functional and clustering analyses and graphical representations were performed using R/Bioconductor (v4.1.0) [51].

### 4.7. Real-Time qPCR

One hundred μL of isolated EVs were incubated with 3.5 ng/μL proteinase K (Thermo Fisher Scientific, Waltham, MA, USA) for 10 min at 37 °C, and neutralized with 17.6 μM of proteinase K inhibitor (Merk, Rahway, NJ, USA) for 10 min at RT. Next, 1 ng/μL RNase A (Thermo Fisher Scientific) was incubated with EVs for 20 min at 37 °C and neutralized with 1.5 U/μL RNaseOUT (Thermo Fisher Scientific) for 5 min at RT. Then, RNA was extracted using ReliaPrep RNA Tissue Miniprep System Kit (Promega, Fitchburg, WI, USA) following the manufacturer’s instructions. Total RNA was reverse transcribed with random primers (Agilent, Santa Clara, CA, USA) and oligo-dT (Agilent) and the AffinityScript multiple temperature reverse transcriptase (Agilent).

Real-time qPCR was performed on the QuantStudio Real time PCR System (Thermo Fisher Scientific) using commercially available qPCR gene expression assays (Integrated DNA Technologies, IDT, Redwood City, CA, USA) for IGFBP-2 (Hs01040719_m1), NECTIN-2 (Hs01071562_m1) and PECAM-1 (Hs01065282_m1). Data were expressed as raw Ct values.

### 4.8. Protein Determination Using ELISA

Serum levels of IGFBP-2 (ab100540, Abcam, Cambridge, UK, lower detection limit 20 pg/mL), PECAM-1 (ELH-PECAM-1, RayBiotech, Peachtree Corners, GA, USA), lower detection limit 15 pg/mL), and NECTIN-2 (ELH-NEC2, RayBiotech, lower detection limit 0.12 ng/mL) were measured using ELISA with intra- and inter-assay coefficients of variation of 10% and <12%, respectively, following the manufacture’s protocols.

### 4.9. Morphological Analysis of Retrieved IS Thrombi and AF Atria

Retrieved thrombi (*n* = 23) were fixed in formaldehyde (PanReac AppliChem, Barcelona, Spain) for 24 h. Then, samples were embedded in paraffin using a tissue automatic processor (Tissue-Tek VIP, Chatsworth, CA, USA) and cut into 3 µm sections with a rotatory microtome (HM-340E, Microm, Walldorf, Germany). Serial slides from each thrombus were stained with hematoxylin and eosin (PanReac AppliChem), and immunochemistry was performed with the following primary antibodies: antihuman IGFBP-2 (1:500 dilution, MA5-15400, Invitrogen), antihuman CD45 (1:100 dilution, NCL-L-LCA, Leica Biosystems, Nussloch, Germany), antihuman CD68 (0.04 µg/mL, M0184, Dako), and neutrophil elastase (5 µg/mL, HPA066836, Sigma). Mouse or rabbit secondary antibody conjugated with horseradish peroxidase (Envision System HRP labeled polymer, Dako) were used as required. IGFBP-2 underwent a preamplification step with the TSA Biotin System (NEL700A001KT, PerkinElmer, Waltham, MA, USA), following the manufacturer’s instructions, before immunoreactivity detection with the DAB system (Dako). Slides were scanned using Aperio CS2 system (Leica Biosystems) and analyzed with Fiji ImageJ Software (National Institute of Health, v1.53c). The brown regions and tissue area were automatically detected using a macro designed for this purpose. Data are presented as the percentage of positive stained area in total tissue area. The immunohistochemistry for IGFBP-2 in human left atrium samples (*n* = 13) of AF patients was performed in 4 µm paraffin-embedded sections using a monoclonal mouse antibody (1:200 dilution, MA5-15400, Invitrogen).

### 4.10. In Vitro Studies

#### 4.10.1. Cell Culture

The THP-1 monocyte cell line obtained from the American Type Culture Collection (ATCC, Virginia, VA, USA) was grown at a density of 2 × 10^6^ cells in a 75 cm^2^ flask in RPMI-1640 medium (Life Technologies, Carlsbad, CA, USA) containing 10% fetal bovine serum (FBS, Sigma-Aldrich), 1% penicillin and streptomycin (PS, 100 U/mL and 100 µg/mL, Sigma-Aldrich), pyruvate (1 mM, Sigma-Aldrich), 10 mM HEPES (Sigma-Aldrich), and 14.3 mM 2-mercaptoethanol (Sigma-Aldrich).

Immortalized human aortic endothelial cells TeloHAEC (ATCC^®^ CRL-4052^TM^) were cultured in vascular cell basal medium (VBM, ATCC PCS-100-030) supplemented with vascular endothelial cell growth kit-VEGF (ATCC PCS-100-041: 5 ng/mL rhVEGF, 5 ng/mL rhEGF, 5 ng/mL rhFGF basic, 15 ng/mL rhIGF-1, 10 mM L-glutamine, 0.75 U/mL Heparin sulfate, 1 µg/mL Hydrocortisone, 50 µg/mL Ascorbic acid and 2% FBS) and 1% penicillin/streptomycin.

The immortalized human brain microvascular endothelial cell line (hCMEC/D3, SC0066, Merck, Branchburg, NJ, USA) was seeded on a 75 cm^2^ flask precoated with 0.7 mg/mL rat collagen type I for 2 h (08-115, Sigma-Aldrich) and cultured in EndoGRO^TM^-MV complete culture media (SCME004, Merck).

All cell lines were maintained in a humidified environment at 37 °C with 5% CO_2_, and the medium was renewed every 2–3 days.

#### 4.10.2. Leukocyte Transendothelial Migration Assay

A total of 150,000 TeloHAECs were seeded into 5 µm-pore 6.5 mm transwells (Merck) in complete VBM. A total of 600 µL of complete VBM were added at the bottom. Cells were allowed to grow for 48 h. Then, the cells were washed with PBS and incubated overnight in serum-free media endothelial cells (SFMEC, 11111-044, GIBCO, Waltham, MA, USA) and 1% PS. To activate the endothelium, TeloHAECs were stimulated with TNFα (20 ng/mL, Sigma) in the presence or absence of a neutralizing antibody against IGFBP-2 (600 ng/mL, AF674, R and D, following the manufacturer’s neutralization dosage recommendation) in SFMEC for 2 h. In the meantime, THP-1 cells were stained with calcein (0.2 mM, Invitrogen) for 30 min at 37 °C. A total of 300,000 calcein-stained monocytes were added on top of stimulated TeloHAECs with or without the specified neutralizing antibody. Monocytes migrated towards the bottom chamber containing 1% FBS, 0.5 % BSA, and 1% PS in RPMI. After 4 h, transmigrated cells were harvested, resuspended in 5% FBS and 2 mM EDTA in PBS and analyzed using flow cytometry on a CytoFlex cytometer (Beckman Coulter). The results were analyzed with CytExpert 2.3 software (Beckman Coulter).

#### 4.10.3. Endothelial Permeability

A total of 60,000 hCMEC/D3 were seeded on top of 6.5 mm transwells with a 3 µm-pore size (Merck) that were precoated with collagen type I and allowed to grow in complete medium for 8 days, changing the medium every 2 days. A total of 600 µL of complete EndoGRO^TM^-MV medium was added at the bottom chamber. After 8 days, the cells were washed with PBS and incubated overnight in SFMEC. The hCMEC/D3 monolayer was then treated with 5 U/mL human thrombin (T4393, Sigma) with or without IGFBP-2 antibody for 2 h, as explained above. To assess hCMEC/D3 permeability, 100 µg/mL BSA-FITC (Invitrogen) was added on top of the monolayer and allowed to permeabilize to the bottom chamber for 0, 15, 30, and 60 min. Fluorescence in the bottom chamber was assessed with 485 nm and 535 nm filters, excitation and emission, respectively, in a fluorescence plate reader (SpectraMax, Gemini XS, Molecular Devices Corporation, San Jose, CA, USA).

### 4.11. Rotational Thromboelastometry (ROTEM)

A total of 300 μL of fresh citrated whole blood from healthy volunteers (*n* = 5) was assessed using rotational thromboelastometry (ROTEM^®^, Tem Innovations GmbH, Munich, Germany) using an EXTEM assay in the presence or absence of anti-IGFBP-2 antibody and in the presence or absence of 300 IU/mL recombinant t-PA (Actilyse, Boehringer-Ingelheim, Rhein, Germany). We recorded clotting time (CT, seconds), clot formation time (CFT, seconds), maximum clot firmness (MCF, mm), clot lysis index at 30 min (CLI30), and lysis onset time (LOT, seconds).

### 4.12. Statistical Analysis

Data normality was assessed using the Shapiro–Wilk test. Differences between more than 2 groups were assessed using ANOVA or Kruskal–Wallis, followed by Bonferroni or Dunn’s test, while comparison between 2 groups was determined using Student’s *t*-test or Mann–Whitney U test, according to data normality. Correlations were performed using Pearson or Spearman tests between two independent variables. Receiver operating characteristic (ROC) curve analyses were used to assess the abilities of the candidates in predicting stroke etiology. The variable with best predictive performance was dichotomized at the cutoff point maximizing Youden’s index and analyzed using multivariable binary logistic regression. We selected variables with *p* ≤ 0.10 in the univariate analysis to enter as covariates into the multivariate model. *p* < 0.05 was considered statistically significant, and only two-sided *p* values were used. We used SPSS v25.0 and GraphPad version 9.0 for data analysis.

## 5. Conclusions

In conclusion, the transcriptomic analysis of circulating EVs from patients with IS might be a valuable tool for the identification of potential diagnostic biomarkers for the etiology of IS, which could contribute to improving the diagnosis and therapeutic strategy for these patients. In particular, IGFBP-2 emerges as a potential biomarker of embolic IS etiology involved in processes intrinsically related to IS pathophysiology, such as clot formation and endothelial dysfunction.

## Figures and Tables

**Table 1:** Demographic and clinical characteristics of all IS patients (*n* = 114) and according to IS etiology.

Patient’s Characteristics	All Patients *n* = 114	AT *n* = 26	CE *n* = 64	ESUS *n* = 24	*p*
Risk factors *n* (%)					
Age, years	75.5 (64.7–82.0)	72.0 (65.7–79.0)	76.0 (64.0–82.0)	73.0 (64.0–83.0)	0.66
Sex, female	50 (43.9)	3 (11.5)	36 (56.3)	11 (45.8)	0.001
Serum glucose at admission (mg/dL)	114.0 (101.0–140.0)	124.0 (99.5–162.5)	110.0 (99.0–130.0)	119.0 (106.25–169.2)	0.27
Smoking	6 (5.3)	1 (3.8)	3 (4.9)	2 (9.5)	0.66
Dyslipidemia	69 (60.5)	19 (76.0)	33 (51.6)	17 (70.8)	0.05
Hypertension	77 (67.5)	25 (96.2)	37 (57.8)	15 (62.5)	0.002
Diabetes	23 (20.2)	5 (19.2)	12 (18.8)	6 (25.0)	0.80
Cardiopathy	38 (33.3)	7 (30.4)	26 (40.6)	5 (20.8)	0.20
Atrial fibrillation	49 (44.1)	1 (4.3)	48 (75.0)	0 (0.0)	<0.001
Previous IS	16 (17.0)	2 (11.1)	11 (19.6)	3 (15.0)	0.67
Therapies *n* (%)					
Antiplatelet therapy	26 (22.8)	11 (42.3)	10 (15.6)	5 (20.8)	0.02
Anticoagulant therapy	28 (24.8)	0 (0.0)	27 (42.9)	1 (4.2)	<0.001
Statins	53 (46.5)	16 (61.5)	24 (37.5)	13 (54.2)	0.08
Fibrinolytic therapy (tPA)	66 (57.9)	16 (61.5)	37 (54.7)	15 (62.5)	0.73
Successful recanalization	104 (92.3)	21 (87.5)	60 (93.8)	23 (95.8)	0.48
Hemorrhagic transformation	39 (35.5)	7 (31.8)	23 (35.9)	9 (37.5)	0.91
Baseline NIHS *n* (%) S					
Mild stroke (<7)	6 (5.4)	3 (11.5)	2 (3.2)	1 (4.3)	0.27
Moderate (7–14)	40 (35.7)	10 (38.5)	23 (36.5)	7 (30.4)	0.82
Severe (>14)	66 (58.9)	13 (50.0)	38 (60.3)	15 (65.2)	0.52
Three-months outcomes *n* (%)					
Functional independence	61 (55.5)	13 (52.0)	36 (57.1)	12 (54.5)	0.90
3-month mortality	23 (20.4)	9 (36.0)	8 (12.7)	6 (27.3)	0.03

Continuous data are presented as median (IQR). Categorical data are presented as number (percentage). The comparisons were performed using Person chi-square or Fisher exact tests for categorical variables and Kruskal–Wallis for quantitative variables. AT = atherothrombotic; CE = cardioembolic; ESUS = embolic stroke from undetermined source; IS = ischemic stroke.

## Data Availability

RNAseq data are available at the GEO repository (GSE255087). The raw data supporting the conclusions of this article will be made available by the authors on request.

## Supplementary Materials

The supporting information can be downloaded at https://www.mdpi.com/article/10.3390/ijms25084379/s1.
