# Supplementary material for: Transcriptomic Analysis of Extracellular Vesicles in the Search for Novel Plasma and Thrombus Biomarkers of Ischemic Stroke Etiologies
Source: Int J Mol Sci. 2024 Apr 16;25(8):4379. doi: 10.3390/ijms25084379 (PMC11050408; doi:10.3390/ijms25084379)
Supplement: Supplementary file 1 [file ijms-25-04379-s001.zip › ijms-2946263-supplementary.pdf]

Supplemental data

Table S1: Demographic and clinical characteristics of patients with IS included in the transcriptomic analysis of plasma EVs (n=21)

Patient's characteristics	All patients n=21	AT n=6	CE n=10	ESUS n=5	p
Risk factors n(%)					
Age, years	76.0(65.5-82.5)	78.5(65.7-80.0)	74.5(63.0-82.9)	70.0(64.0-83.0)	0.92
Sex, female	10(47.6)	1(16.6)	7(70)	2(40)	0.10
Smoking	2(15.3)	0(0.0)	1(11.1)	1(25)	0.45
Dyslipidemia	11(52.3)	5(83.3)	2(20)	4(80)	0.01
Hypertension	13(61.9)	6(100)	4(40)	3(60)	0.05
Diabetes	2(9.5)	2(33.3)	0(0.0)	0(0.0)	0.06
Atrial fibrillation	10(47.6)	1(16.7)	9(90.0)	0(0.0)	0.001
Previous IS	2(9.5)	1(16.6)	1(10.0)	0(0.0)	0.64
Therapies n(%)					
Antiplatelet therapy	4(19.0)	3(50)	0(0.0)	1(20.0)	0.04
Anticoagulant therapy	4(19.0)	0(0.0)	3(30.0)	1(20.0)	0.33
Statins	10(47.6)	5(83.3)	1(10.0)	4(80.0)	0.004
Fibrinolytic therapy (tPA)	12(57)	3(50.0)	5(50.0)	4(80.0)	0.49
Successful recanalization	20(95.2)	5(83.3)	10(100.0)	5(100.0)	0.23

Baseline NIHSS n(%)					
Moderate (7-14)	8(38.0)	3(50.0)	4(40.0)	1(20.0)	0.58
Severe (>14)	13(62.0)	3(50.0)	6(60.0)	4(80.0)	0.58
Three-months outcomes n(%)					
Functional independence	9(42.8)	2(33.3)	5(50.0)	2(40.0)	0.49
3-month mortality	5(23.8)	3(50.0)	1(10.0)	1(20.0)	0.11

Continuous data presented as median (IQR). Categorical data are presented as number (percentage). The comparisons were performed using Person chi-square for categorical variables, and Kruskal-Wallis for quantitative variables). AT=atherothrombotic; CE=Cardioembolic; ESUS=Embolic Stroke from Undetermined source; IS=Ischemic stroke.

Table S2: Differentially expressed genes (DEG) after the transcriptomic analysis of circulating EVs of IS patients (AT n=4-6, CE n=6-10, ESUS n=4-5).

ENSEMBL ID	NAME	FOLD CHANGE	AVEEXPR (LOG2CPM)	p (LIMMA VOOM)
AT vs others (n=14)				
ENSG00000130202.10	NECTIN-2	4.2	2.6	0.001
CE vs others (n=21)				
ENSG00000115457.10	IGFBP-2	2.3	2.0	0.01
ENSG00000261371.6	PECAM-1	2.3	3.9	0.01

Fold change between the different contrasts are shown (AT vs. others, and CE vs others), as well as the average gene expression in all the samples (AveExpr, Log2CPM)

Table S3: Association of NECTIN-2 and IGFBP-2 circulating levels with clinical characteristics and outcome in patients with IS.

	NECTIN-2 (ng/mL) n=90			IGFBP-2 (ng/mL) n=81		
Patient's characteristics	No	Yes	p	No	Yes	p
Risk factors						
Sex, female	1.6(1.09-2.5)	2.0(1.1-4.5)	0.11	333.5(213.2-684.9)	346.6(237.3-646.2)	0.67
Hypertension	1.7(1.09-2.7)	1.8(1.2-3.3)	0.48	314.8(206.8-535.7)	387.2(226.8-682.4)	0.35
Diabetes Mellitus	1.7(1.1-2.6)	2.7(1.4-4.1)	0.14	329.9(213.0-646.2)	398.8(267.1-681.6)	0.57
Dyslipidemia	2.3(1.2-4.0)	1.6(1.0-2.9)	0.10	389.6(220.4-744.2)	325.1(213.4-633.7)	0.43
Smoking	1.7(1.1-3.2)	2.0(1.2-5.0)	0.59	329.9(213.4-621.2)	925.9(275.7-1373.2)	0.21
Cardiopathy	1.7(1.07-2.4)	2.3(1.3-3.7)	0.09	323.0(212.9-649.4)	378.2(267.1-777.3)	0.21
Atrial fibrillation	1.7(1.0-2.6)	2.0(1.4-4.1)	0.12	329.6(203.2-633.7)	559.4(298.4-891.4)	0.02
Previous IS	1.7(1.2-2.7)	2.6(1.3-4.7)	0.09	338.1(214.4-669.1)	349.0(231.9-527.2)	0.77
Therapies						
Peripheral arteriopathy	1.7(1.1-3.2)	1.7(1.4-3.2)	0.99	343.7(213.8-651.0)	326.3(234.2-517.2)	0.73
Antiplatelet therapy	1.7(1.0-3.0)	1.7(1.4-3.5)	0.39	346.6(237.3-662.8)	335.3(203.2-554.8)	0.27
Anticoagulant therapy	1.7(1.0-3.0)	1.7(1.4-3.5)	0.44	329.9(209.0-633.3)	554.7(257.2-1080.5)	0.08
Statins	2.0(1.2-3.7)	1.6(1.0-2.6)	0.09	357.1(246.0-738.1)	327.3(212.4-639.9)	0.23
Fibrinolytic therapy (tPA)	1.7(1.0-2.7)	1.7(1.3-3.2	0.28	340.9(226.0-632.3)	338(203.6-679.1)	0.94

Successful recanalization	1.6(1.2-1.9)	1.7(1.1-3.0)	0.47	546.2(226.1-667.0)	335.4(213.8-651.0)	0.86
Hemorrhagic transformation	1.8(1.1-2.8)	1.7(1.1-4.0)	0.74	340.9(237.9-650.3)	346.6(212.7-652.6)	0.86
Baseline NIHSS						
Minor Stroke (<7)	NA	1.3(0.9-6.4)		293.5(143.7-403.1)	NA	
Moderate (7-14)	NA	1.8(1.4-2.4)	0.98	323.7(201.7-651.0)	NA	0.15
Severe (>14)	NA	1.7(1.0-3.9)		430.1(280.7-723.2)	NA	
Three-months outcomes n(%)						
3-month mortality	1.7(1.1-3.1)	2.1(1.5-2.8)	0.42	327.5(212.9-646.2)	482.6(313.9-897.2)	0.19
Functional dependence	1.6(1.1-3.0	2.0(1.4-3.1)	0.21	327.2(204.4-620.5)	478.4(304.0-779.7)	0.03

Values presented as median and IQR. NA: not applicable. The comparisons were made using Mann-Whitney U or Kruskal Wallis test for NECTIN-2 and student t test or ANOVA for log transformed values of IGFBP-2.

Table S4. Univariate and multivariate logistic regression models for IS etiology (CE and ESUS, n=61 vs AT, n=17) and categorized IGFBP-2 (cut-off >247.6 ng/mL).

	Univariate		Multivariate model 1		Multivariate model 2	
Variable	OR (95%CI)	p	OR (95%CI)	p	OR (95%CI)	p
IGFBP-2	5.5 (1.7-16.8)	0.003	9.5(2.13-42.54)	0.003	8.7(1.84-41.13)	0.003
Age, years			1.0(0.98-1.09)	0.200	1.0(0.98-1.09)	0.208
Sex, female			15.3(2.71-87.34)	0.002	14.6(2.55-83.62)	0.003
NIHSS					1.0(0.88-1.09)	0.704

OR= Odds ratio; CI= confidence interval; NIHSS= National Institutes of Health Stroke Scale.

Table S5: Demographic and clinical characteristics of hypertensive patients with and without atrial fibrillation (AF).

Patient's characteristics	Hypertensive patients without AF n=20	Hypertensive patients with AF n=78
Age, years	58.0 (53.3-60.8)	68.0 (60.0-73.0)
Sex, female	11 (55)	20 (25.6)
Dyslipidemia	12 (60.0)	45 (57.7)
Diabetes	0 (0.0)	10 (12.8)
Cardiopathy	0 (0.0)	4 (5.1)
Anticoagulant Therapy	0 (0.0)	70 (89.7)

Continuous data presented as median (IQR). Categorical data are presented as number (percentage).

Table S6. Association of thrombi IGFBP-2 expression and clinical characteristics in patients with IS (n=23).

	% of IGFBP-2 area/ total area		
Patient's characteristics	No	Yes	p
Risk factors			
Sex, female	3.3(2.8-3.7)	4.3(3.1-6.4)	0.35
Hypertension	5.1(3.6-8.6)	3.0(2.2-3.4)	0.009
Diabetes Mellitus	3.7(2.8-5.7)	3.1(3.0-3.3)	0.58
Dyslipidemia	4.1(2.4-6.6)	3.3(2.9-5.2)	0.94
Smoking	3.4(2.7-4.7	3.7(3.3-4.1)	0.88
Therapies			
Antiplatelet therapy	3.9(3.1-6.4)	3.0(2.9-3.1)	0.31
Anticoagulant therapy	3.3(2.8-3.7	8.6(8.5-8.8)	0.03
Statins	4.1(3.3-5.7)	3.2(2.7-4.7)	0.23
Baseline NIHSS			
Moderate (7-14)	NA	3.4(3.2-4.5)	0.68
Severe (>14)	NA	3.3(2.8-4.1)	
Successful recanalization	NA	3.5(2.9-5.4)	NA
Three-months outcomes			
3-month mortality	3.9(3.4-5.1)	3.1(2.4-5.9)	0.05
Functional dependence	3.7(3.1-4.7)	3.7(2.4-8.6)	0.70

Data are presented as median (IQR). NA: not applicable. The comparisons were made using Mann-Whitney U test.

Supplementary Figures

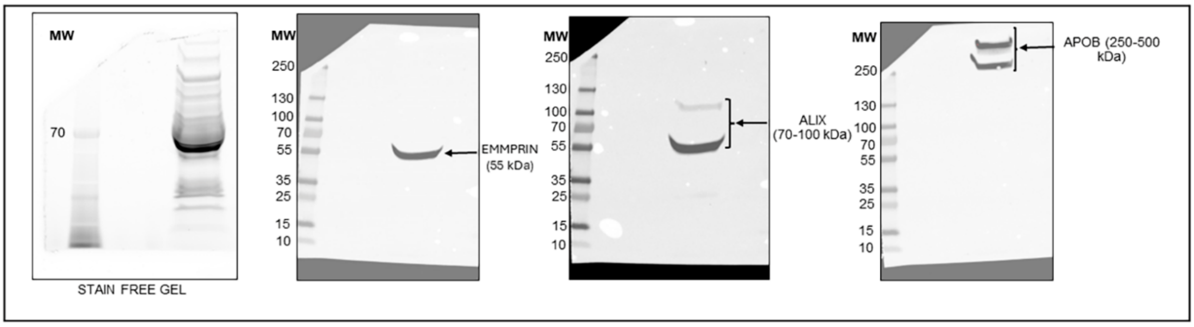

Figure S1: Western blot for the EVs markers EMMPRIN and ALIX, and plasma contaminant (APOB100) in EVs from plasma.

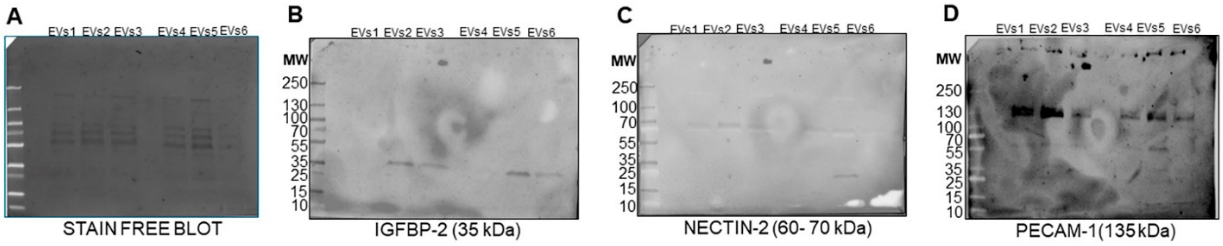

Figure S2: Western blot of plasma EVs from IS patients (n=3 AT and 3 CE) showing selected candidates and their expected molecular weights (MW). EVs 1 to 3 are from AT patients and 4 to 6 are from CE patients.

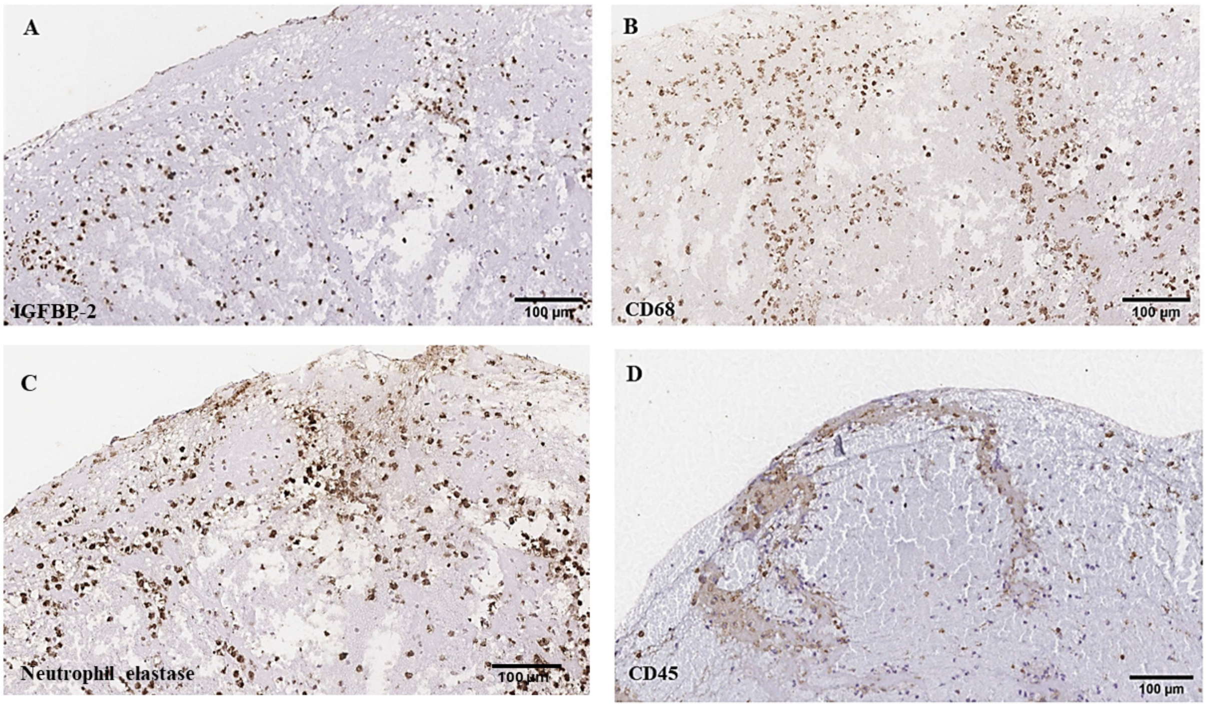

Figure S3: Representative serial images of CE thrombus immunostained for IGFBP-2 (A), CD68 - labelling macrophages (B), Neutrophil Elastase - labelling neutrophils (C), and CD45 - labelling leukocytes (D). Scale bars represent 100 µm.

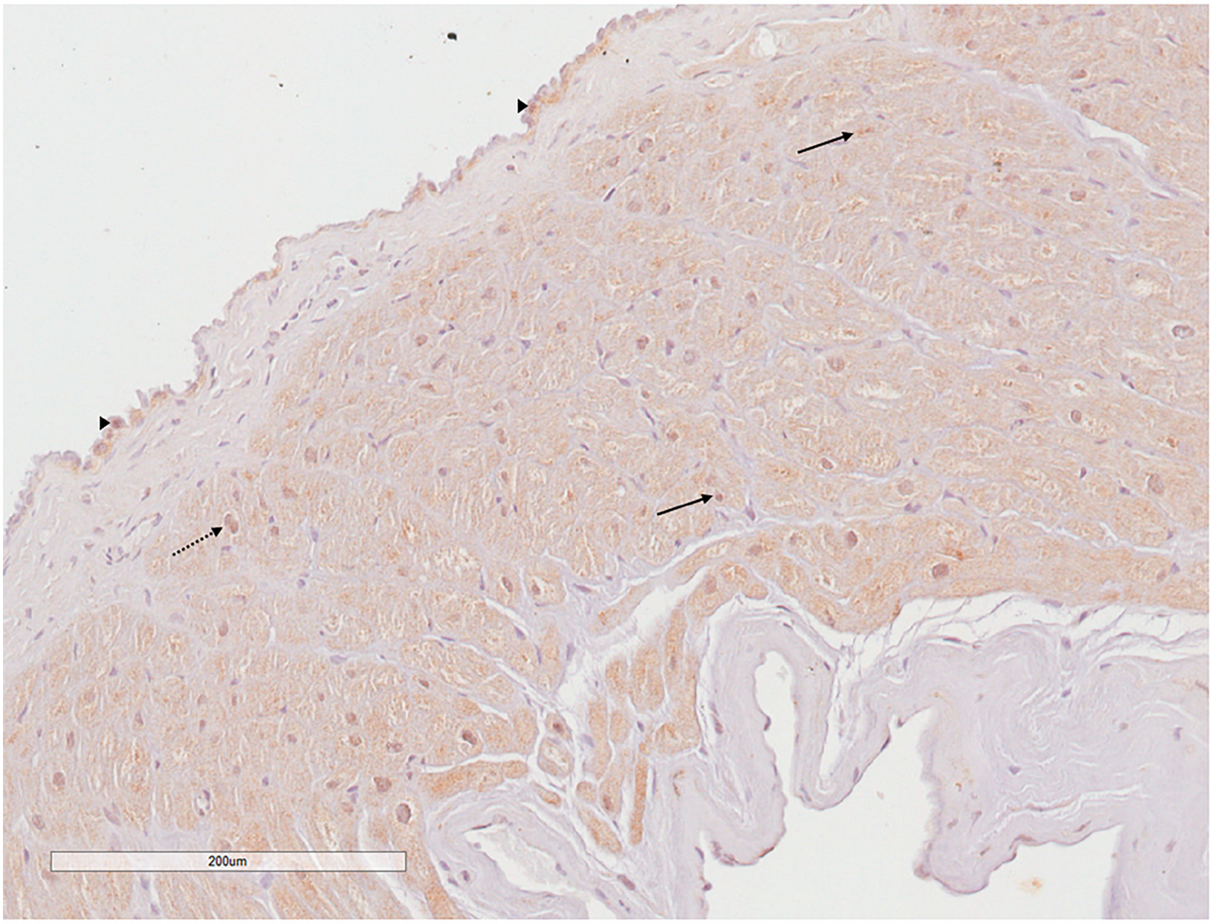

Figure S4: Immunostaining of IGFBP-2, in the left atrium of patient with AF. IGFBP-2 was found mostly in cardiomyocytes (arrow), in endothelial (dotted arrow), and mesothelial (arrowhead) cells. Scale bar represent 200 µm.
